# Pharmacokinetics of metformin in patients with chronic kidney disease stage 4 and metformin‐naïve type 2 diabetes

**DOI:** 10.1002/prp2.424

**Published:** 2018-09-14

**Authors:** Ajith M. Dissanayake, Mark C. Wheldon, Christopher J. Hood

**Affiliations:** ^1^ Counties Manukau Health Auckland New Zealand; ^2^ Auckland University of Technology Auckland New Zealand; ^3^ Middlemore Clinical Trials Middlemore Hospital Auckland New Zealand

**Keywords:** chronic kidney disease, diabetes mellitus, metformin, pharmacokinetics, phase I trial, single‐compartment model

## Abstract

The pharmacokinetics of metformin therapy in patients with chronic kidney disease stage 4 (CKD‐4) were studied using data from the largest Phase I consecutive cohort trial yet performed in this population. Eighteen metformin‐naïve men and women with Type 2 Diabetes and creatinine clearance (CrCl) in the range 18‐49 mL/min (eGFR 15‐29 mL/min/1.73 m^2^) were allocated to daily immediate‐release metformin of 250 mg, 500 mg, or 1000 mg. A first‐dose profile and trough concentrations for 4 weeks were taken on all patients. Pharmacokinetic (PK) parameters were estimated by fitting a first‐order compartment model with absorption in a peripheral compartment to concentrations measured 24 hours post–first dose. Single‐dose PK parameters time to maximum concentration (*t*_max_) and maximum concentration (*C*_max_) were consistent with previous observations in patients with normal renal function (healthy and diabetic), as was the association between CrCl and apparent total oral clearance (*Cl/F*). However, patients with a CrCl below 32 mL/min had trough concentrations that were consistently above the steady‐state minimum implied by the population PK model. This suggests the model may not apply to patients with CrCl below 32 mL/min. Metformin in doses of 500‐1000 mg/day could be taken by CKD‐4 patients. However, the single‐compartment model breaks down as CrCl declines below 32 mL/min suggesting that metformin levels should be monitored regularly in progressive stage 4 CKD.

**Abbreviations**
ABW: adjusted body weight
AIC: Akaike's information criterion
BIC: Bayesian information criterion
BLUPs: best linear unbiased predictors
BMI: body mass index
CIs: confidence intervals
CKD‐4: chronic kidney disease stage 4
eGFR: estimated glomerular filtration rate
IBW: ideal body weight
IQR: interquartile range
popPK: population pharmacokinetics
TBW: total body weight

## 1 INTRODUCTION

Metformin, a time tested medication, is known to lower mortality and morbidity in patients with type 2 diabetes mellitus (T2DM).^1^ Metformin is predominantly cleared renally^2^ and many patients with chronic kidney disease (CKD) are not offered this medication as there is a perceived risk of life‐threatening lactic acidosis secondary to metformin toxicity. Lactic acidosis is the presence of metabolic acidosis (pH < 7.35, bicarbonate <22 mmol/L) in the setting of hyperlactatemia (lactate ≥ 5 mmol/L). However, this perceived risk is largely based on case reports^3^ and the fact that phenformin, a precursor of metformin of the same therapeutic class but with different mechanisms of action, undoubtedly did cause significant rates of lactic acidosis.^4^

Many large observational studies have failed to find evidence of metformin‐associated lactic acidosis. Scale and Harvey^5^ reviewed all cases of lactic acidosis in a large Welsh hospital from December 2005 to June 2009. They identified 149 cases of which 48 had T2DM. Of the 28 taking metformin 18 were Cohen and Woods,^6^ Class B and had mean estimated glomerular filtration rate (eGFR) = 20.4 mL/min/1.73 m^2^. They found no evidence for an effect of metformin on lactic acidosis. A Cochrane review^7^ studied lactic acidosis in patients with T2DM. They identified 143 prospective studies comprising 37 360 patient‐years of metformin use that did not exclude patients with renal insufficiency (creatinine level ≥133 μmol/L). No reported cases of fatal or nonfatal lactic acidosis were found suggesting the risk in CKD patients could be low. A systematic review by Inzucchi et al^8^ identified 65 studies covering cohorts with eGFR in the range 30‐60 mL/min/1.73 m^2^ and concluded that a change in metformin prescribing guidelines is worth investigating for patients with CKD up to stage 3B (eGFR 30 to <45 mL/min/1.73 m^2^). Hung et al^9^ conducted a retrospective observational cohort study of patients with T2DM and CKD stage 5 (CDK‐5; serum creatinine > 530 μmol/L) in Taiwan from January 2000 to December 2009. They performed a matched comparison between 813 metformin users and 2439 nonusers and found that metformin use increased the risk of all‐cause mortality but not lactic acidosis. Patients with CKD‐5 were excluded from our study. Inzucchi et al^8^ and Hung et al^9^ called for more studies to investigate the safety and therapeutic effect of metformin therapy in patients with CKD.

Despite the literature cited above, use of metformin in this population remains controversial^10^, ^11^ and contrary to national guidelines in many jurisdictions.^8^ However, small studies^12^ and ones reporting only steady‐state concentrations^13^, ^14^ show that therapeutic levels should be achievable. A simulation study by Duong et al^15^ based on observational data including CKD patients reached the same conclusion. A recent consecutive dose‐escalating study of metformin in patients with metformin‐naïve T2DM and CKD stage 4 (CKD‐4; eGFR 15‐30 mL/min/1.73 m^2^) by Dissanayake et al^16^ found no episodes of hyperlactatemia or metabolic acidosis and no significant change in any biochemical safety measures and argued for liberalization of metformin use in this population. Here, we report the pharmacokinetic (PK) results of that study and assess the impact of kidney function on single‐dose PK profiles.

## 2 MATERIALS AND METHODS

### 2.1 Subjects and protocol

Eligibility criteria and study protocol are described elsewhere.^16^ Briefly, metformin‐naïve men and women aged 30‐75 years weighing <160 kg with T2DM were eligible for this study if their diabetes had been diagnosed at least 2 years prior to screening (in accordance with American Diabetes Association criteria), their HbA1c was in the range 42.1‐96.7 mmol/mol (6‐11%), and their eGFR in the range 15‐29 mL/min/1.73 m^2^. Diabetes duration was determined from first laboratory diagnosis or patient report if that was not available. Creatinine clearance (CrCl) was not available at screening hence the use of eGFR to indicate kidney function. CrCl for the study cohort recruited was in the range 18‐49 mL/min. Patients having their T2DM treated with diet, oral hypoglycemic medication, or insulin were accepted. Among the excluded were those previously treated with metformin or with a demonstrated metformin intolerability with chronic kidney disease stage 1, 2, 3, or 5 (eGFR ≥ 30 mL/min/1.73 m^2^ or <15 mL/min/1.73 m^2^) and those currently receiving renal replacement therapy (hemodialysis chronic ambulatory peritoneal dialysis) or renal transplant.

This was a Phase I open‐label consecutive group dose‐escalating study with follow‐up of 32 days. The total study size was 18 patients. Three consecutive cohorts (1, 2, and 3) of 6 patients each were recruited to receive 250, 500, or 1000 mg once‐daily doses of metformin, respectively. A total of 8 patient visits were scheduled at Days 1, 4, 5, 11, 18, 25, 29, and 32 with baseline information collected at Visit 1 (Day 1). The intervention consisted in a single dose of metformin in an immediate‐release tablet taken orally before breakfast after an overnight fast. On visit days the drug was to be taken after the visit prior to the first meal. Visits were held in the morning.

A consecutive cohort design was chosen because this was a safety and tolerability Phase I study (this group had not been prescribed metformin) with pharmacokinetic evaluation on different doses. The study size was chosen to be consistent with other pharmacokinetic studies in the nonrenal population; no a priori power calculation was performed.

The New Zealand Health and Disability Ethics Committee approved this study (reference number NTX/11/12/112) and all participants gave written informed consent prior to enrolment. Safety monitoring (including for signs of acidosis) was done by an independent physician.

### 2.2 Outcomes

The primary safety outcome of the trial was the development of acidosis assessed via fasting levels of venous lactate, bicarbonate, and pH. This was reported on in Dissanayake et al.^16^ In this study we assess additional outcomes, namely, single‐ and repeat‐dose fasting serum metformin concentrations. Each participant's first dose was taken at Visit 2 (Day 4) after baseline serum metformin levels were taken. Metformin levels were then taken at 2, 4, 6, 8, and 24 hours (Visit 3) post–first dose. Pharmacokinetic parameters were determined using these concentrations which totaled 102 observations (5‐6 per patient over a 24‐hour period). Pre–dose concentrations were taken at all subsequent visits (Visits 4‐8). Patients were instructed to fast overnight and present for their laboratory test in the morning prior to taking that day's dose.

### 2.3 Other measures

Estimated glomerular filtration rate was calculated from serum creatinine (*Cr*), gender, age (in years), and ethnicity using the following formula:

$$\text{eGFR}=141\cdot {\left(\min\left(\frac{\mathrm{Cr}}{a},1\right)\right)}^{b}{\left(\max\frac{\mathrm{Cr}}{a},1\right)}^{-1.209}\cdot {\left(0.993\right)}^{\mathrm{age}}\cdot c\cdot k$$

The constants *a, b,* and *c* are gender‐specific parameters with values in Table 1. The constant *k* had the value 1.159 for patients of Māori or Samoan ethnicity and 1 for all other patients.

**Table 1:** Values of gender‐specific constants used in calculation of eGFR

Constant	Gender	Value
a	M	79.6
	F	61.9
b	M	−0.411
	F	−0.329
c	M	1
	F	1.018

Creatinine clearance (CrCl) was estimated using the modified Cockroft‐Gault equation with total body weight (TBW) if body mass index (BMI) < 18.5 kg/m^2^, ideal body weight (IBW) if BMI 18.5‐22.9 kg/m^2^, and adjusted body weight (ABW) if BMI ≥ 23 kg/m^2^. ABW was defined as IBW + 0.4 × (TBW‐IBW).^18^, ^19^, ^20^ Our calculations are shown in Table A1. All patients had BMI ≥ 23 kg/m^2^.

### 2.4 Statistical analysis

#### 2.4.1 Methods

Basic descriptive statistics were used to summarize patient demographics and baseline status. More complex methods (described below) were used to characterize population pharmacokinetics.

All statistical analysis was done using the *R Environment for Statistical Computing* version 3.1.2.^21^ Nonlinear modeling was done using the *MASS,* 22 *car*,^23^ and *nlme* 24 packages; plots were generated using the *ggplot2* package.^25^ The 0.05 level of significance was used for all statistical tests.

#### 2.4.2 Pharmacokinetics: single‐dose hourly concentrations

Population pharmacokinetic (popPK) parameters were estimated using a nonlinear mixed effects model.^26^ Following Bardin et al^27^ and Duong et al^15^ a first‐order compartment model with absorption in a peripheral compartment was used to relate serum metformin concentration to hours postdose. The model is defined as

(1) $$c\left(t\right)=\frac{{\text{DKk}}_{a}}{\left(Cl/F\right)\left(K-k_{a}\right)}\left(e^{-Kt}-e^{-k_{a}t}\right),$$

where *D* is the dose in mg, *t* is time postdose in hours, and *k*_*a*_ and *K* are the absorption and elimination rate constants, respectively. *Cl/F* is the apparent total oral clearance, where *F* is the relative bioavailability of the drug (0 < *F *≤* *1). Mathematically, *Cl/F* is equivalent to the ratio of the dose to the area under the concentration‐time curve (AUC_0–∞_).^28^ *Cl/F* and *k*_*a*_ were fitted as patient‐specific random intercepts. Actual sampling times were used for all pharmacokinetic evaluations.

Maximum concentration (*C*_max_), time to *C*_max_ (*t*_max_), AUC_0–∞_, and absorption and elimination half‐lives ($t_{1/2}^{k_{a}},t_{1/2}^{K}$, resp.) were derived from the nonlinear mixed effects model estimates of the parameters in Equation (1). Comparison of observed and fitted values and examination of standard residual diagnostics showed the model was a good fit to the data.

Concentration as a function of both dose and CrCl (L/h) was modeled by modifying Equation (1) as follows:

(2) $$c\left(t\right)=\frac{{\text{DKk}}_{a}}{\left(CrCl\right){\left(Cl/F\right)}^{\prime }\left(K-k_{a}\right)}\left(e^{-Kt}-e^{-k_{a}t}\right)$$

where CrCl is the baseline measurement. The factor *(CL/F)ʹ* was fitted as a patient‐specific random effect and is equivalent to *Cl/F* in Equation (1) divided by patient‐specific baseline CrCl. This model was used to generate predicted *C*_max_ values for future patients with a range of baseline CrCls. We used Akaike's Information Criterion (AIC) and the Bayesian Information Criterion (BIC) to compare models (1) and (2).

#### 2.4.3 Pharmacokinetics: repeat dose and trough concentrations

We used the following formulae for minimum, mean, and peak concentrations under repeat dosing (*c*_min,ss_, *c*_avg*,*ss_, and *c*_max,ss_, respectively)^28^, ^29^:

$$c_{\min,\mathrm{ss}}\left(t\right)=\frac{{\text{DKk}}_{a}}{\left(Cl/F\right)\left(K-k_{a}\right)}\left(\frac{e^{-k\tau }}{1-e^{-K\tau }}-\frac{e^{-k_{a}\tau }}{1-e^{-k_{a}\tau }}\right)$$

$$c_{\mathrm{avg},\mathrm{ss}}\left(t\right)=\frac{D}{(Cl/F)\tau }$$

$$c_{\max,\mathrm{ss}}\left(t\right)=\frac{{\text{DKk}}_{a}}{\left(Cl/F\right)\left(K-k_{a}\right)}\left(\frac{e^{-Kt_{\mathrm{mas},\mathrm{ss}}}}{1-e^{-K\tau }}-\frac{e^{-k_{a}t_{\max,\mathrm{ss}}}}{1-e^{-k_{a}\tau }}\right)$$

where *τ* = 24 hours is the dosing interval and

$$t_{\max,\mathrm{ss}}=\frac{1}{k_{a}-K}\text{In}\left(\frac{k_{a}\left(1-e^{-K\tau }\right)}{K(1-e^{-k_{a}\tau })}\right)$$

Confidence intervals for average *c*_min,ss_, *c*_avg,ss_, and *c*_max,ss_ and average values of each under specific baseline *CrCl*s were formed by applying the delta method to the parameter estimates produced by models in Equations (1) and (2).

#### 2.4.4 Observed trough concentrations

Observed trough concentrations were compared with confidence intervals for average *c*_min,ss_, *c*_avg,ss_, and *c*_max,ss_ implied by the popPK model fitted to the single‐dose data (entire data set). The intervals were computed by applying the delta method to the variance‐covariance matrix of the estimated fixed effects regression coefficients and applying the relevant quantiles of the standard normal distribution. They represent a 95% confidence interval for the average value of the response for a given set of values for the explanatory variables.

It is possible that on some visits some patients violated protocol and took metformin before having their predose blood test. These would be suspected if one or two observed trough‐level concentrations per patient were within the 95% confidence interval for the steady‐state maximum concentrations while the patient's other observations were within the confidence interval for the minimum. Values meeting these criteria were identified and labeled in plots. However, because there is no independent way to assess whether or not this occurred no data points were removed from any statistical analyses. It is likely therefore that the within‐ and between‐subjects variances are overestimated (leading to wider confidence intervals) in regression models for the observed trough concentrations. This is a conservative approach.

In exploratory analysis, log concentrations were modeled as a function of dose, CrCl, and BMI. A linear mixed effects model with random intercepts for patient was used to account for repeat observations on each patient. Starting with a model with all two‐way interactions, nonsignificant terms were eliminated sequentially beginning with interactions. All but the main effects of CrCl and dose were significant and only these two explanatory variables were retained.

### 2.5 Metformin assay

A high‐performance liquid chromatographic assay was used to measure metformin concentration in plasma. This was described and validated by Zhang et al^30^ The limit of quantification was approximately 20 μg/L and the coefficient of variation estimated by Zhang et al^30^ from intra‐ and interday assay variance was <9.0%.

### 2.6 Materials

We used generic metformin (active ingredient metformin hydrochloride) which was prescribed by the conducting clinician to each patient. Clinicians obtained metformin from their usual pharmacies. Information about metformin supply in New Zealand is available from New Zealand Medicines and Medical Devices Safety Authority.^31^

## 3 RESULTS

### 3.1 Patient characteristics baseline analysis and safety monitoring

Eighteen patients completed the study. Baseline values for demographic and clinical variables are summarized in Dissanayake et al^16^ Briefly, these were as follows for the entire study cohort expressed as median followed by interquartile range (IQR) in parentheses: age 66.0 (6.54) years; body mass index 38.0 (9.87) kg/m^2^; duration of diabetes 15.0 (7.75) years; eGFR 21.0 (8.0) mL/min/1.73 m^2^; HbA1c 67.5 (25.75) mmol/mol (8.3% IQR: 4.5%); venous pH 7.3 (0.05); serum lactate 1.05 (0.58) mmol/L; and CrCl 30.2 (8.6) mL/min. Two patients in the 250 mg group and 2 in the 1000 mg group were females the remainder were males. No signs of lactic acidosis were observed.

### 3.2 Pharmacokinetic modeling

#### 3.2.1 Single dose

There were 102 concentrations (5‐6 per patient) available for PK analysis recorded over 24 hours after the first single dose. All post–baseline measurements were above the minimum quantifiable amount. Maximum observed concentrations, AUC_0‐∞_, and time to maxima are reported elsewhere.^16^

Pharmacokinetic parameter estimates and summaries of best linear unbiased predictors (BLUPs) are in Table 2. The fixed effects and their confidence intervals are for the average over a population of patients with mean CrCl and *k*_*a*_. The residual standard error (SE) is an estimate of the within‐subject variation on the scale of the response (mg/L). The width of the confidence intervals (CIs) for the fixed effects depends primarily on the magnitude of within‐subject variation. Random effect standard deviations (SDs) estimate the between‐subject variability in *Cl/F* and *k*_*a*_ on their respective scales (L/h and h^−1^, respectively). BLUPs are model‐based estimates of popPK parameters for the patients in the study cohort. BLUP summaries (medians and ranges) are given for parameters with random effects. The width of BLUP ranges depends primarily on the magnitude of the between‐subjects variation.

**Table 2:** Compartmental pharmacokinetic parameters of metformin administered in daily doses of 250 mg, 500 mg, and 1000 mg to patients with CKD‐4

Parameter	Unit	Dose (mg)	Fixed effects^a^		BLUP summaries^b^	
			Estimate	95% CI^c^	Median	Range
*K*	1/h	—	0.119	(0.11, 0.13)		
*k*_*a*_	1/h	—	0.65	(0.45, 0.86)	0.56	(0.05, 1.57)
$t_{1/2}^{K}$	h	—	5.84	(5.22, 6.46)		
$t_{1/2}^{k_{a}}$	h	—	1.06	(0.73, 1.39)		
Cl/F	L/h	—	29.6	(23.8, 35.4)	29.3	(10.7, 47.9)
V/F	L	—	249.7	(201.7, 297.8)		
*t*_max_	h	—	3.19	(2.58, 3.80)		
*C*_max_	mg/L	250	0.69	(0.59, 0.78)		
		500	1.37	(1.18, 1.57)		
		1000	2.74	(2.35, 3.13)		
AUC_0‐∞_	mg h/L	250	8.43	(6.81, 10.06)		
		500	16.87	(13.62, 20.12)		
		1000	33.74	(27.23, 40.24)		
			Random effects			
sd(Cl/F)^d^	L/h	—	11.56	(7.77, 17.17)		
sd(k_a_)^d^	1/h	—	0.395	(0.222, 0.705)		
cor(Cl/F,k_a_)^d^		—	0.785	(0.442, 0.928)		
Resid. SE^e^	mg/L	—	0.095	(0.081, 0.113)		

a Fixed effects estimates and CIs are for the average value over a population of patients meeting eligibility criteria and with average *Cl/F* and *k*_*a*_. Variation in population averages is typically lower than variation in observations on individual patients.

b BLUPs are best linear unbiased predictors for the study cohort. They indicate the variation in observed values for the study cohort. They are only given for the random effects *Cl/F* and *k*_*a*_.

c Confidence intervals for $t_{1/2}^{K},t_{1/2}^{k_{a}}$, *V/F, t*_max,_ *C*_max_ and AUC_0‐∞_ were computed using the delta method.

d Standard deviation of the patient‐specific random effects for Cl/F and *k*_*a*_ and their correlation.

e Within‐patient standard error of metformin concentration.

Mean concentrations estimated from the fixed effects component are shown in Dissanayake et al^16^ (Figure 3A) along with observed concentrations. A clear dose‐proportional response is evident in the fitted concentrations and the dose‐specific parameter estimates *C*_max_ and AUC_0‐∞_.

Note that the *C*_max_ and *t*_max_ values reported in Dissanayake et al^16^ (Table 2) are empirical medians, whereas the values in Table 2 in this article are estimates (with confidence intervals) from the compartment model.

#### 3.2.2 Repeat dose

Trough concentrations were observed 24 hours postdose, the first observation being 7 days (24 hours) after commencing medication. The mean elimination half‐life $\left(t_{1/2}^{K}\right)$ estimated from the single‐dose popPK model was 5.8 hours (95% CI: [5.2, 6.5]; Table 2) suggesting steady state would have been reached in approximately 29 hours. The estimated time to peak concentration at steady state (*t*_max_) implied by the popPK model fitted to the single‐dose data was 3.19 hours (95% CI: [2.58, 3.80]).

**Figure 1:**
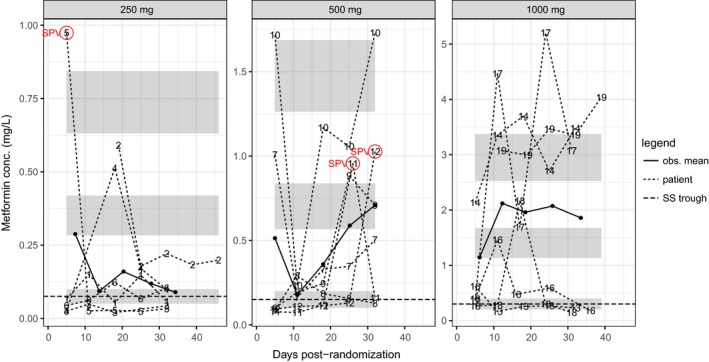
Repeat‐dose serum metformin concentrations recorded at Visits 3‐6 for each dose group 24 hours postdose (trough levels). Number labels and dashed lines indicate observed value; from top to bottom gray bands show the 95% CIs for the means of the theoretical steady‐state maximum mean and minimum concentrations, respectively, for a population meeting eligibility criteria with average Cl/F and *k*_*a*_. SPV, suspected protocol violation; some patients may have erroneously taken their daily dose before the blood test instead of after it. Note that different vertical scales are used in each panel to allow the plots to be easily read

Model‐based estimates of average serum concentrations at steady state were derived from this single‐dose popPK model. Confidence intervals for these parameters have the same interpretation as those for the PK parameters (see above). Estimated population averages for *c*_min,ss_, *c*_avg,ss_, and *c*_max,ss_ are in Table 3. These are plotted along with observed trough concentrations in Figure 1. There is a high degree of between‐patient variation in the observed trough levels. Empirical mean concentrations are not within the confidence intervals for the steady state minimum estimated from the popPK model except perhaps for the 250 mg group. It was suspected that this was due to between‐patient variation in kidney function.

**Table 3:** Estimated average repeat‐dose serum concentrations (mg/L) and 95 percent confidence intervals for the study cohort based on the popPK model in Table 3, and for the healthy cohort of Timmins et al.^17^

Dose (mg/day)	Cohort	*c*_min,ss_ (mg/L)		*c*_avg,ss_ (mg/L)		*c*_max,ss_ (mg/L)	
250	CKD4^a^	0.08	(0.05, 0.10)	0.35	(0.28, 0.42)	0.74	(0.63, 0.84)
500	CKD4^a^	0.15	(0.10, 0.20)	0.70	(0.57, 0.84)	1.47	(1.26, 1.69)
	Healthy^b^			0.35	SD = 0.06	0.65	SD = 0.11
1000	CKD4^a^	0.30	(0.20, 0.40)	1.41	(1.13, 1.68)	2.95	(2.53, 3.37)

a Estimated from this study cohort.

b Reported in Timmins et al^17^ for 250 mg immediate‐release pill given twice daily; 95% CIs were not given.

Consistent with this hypothesis was the finding that the coefficient for CrCl was significant in a regression of baseline CrCl on log trough concentrations, controlling for dose and repeated measures (β = −0.87 95% CI: [−1.68, −0.06]; Table A2). On average, a 1 L/h increase in CrCl corresponded to a reduction in observed metformin trough concentration by a factor of exp(−0.87) = 0.42 (95% CI [0.17, 0.94]). We investigated the impact of kidney function on single‐ and repeat‐dose pharmacokinetics by fitting a second popPK model.

### 3.3 Pharmacokinetics and kidney function

#### 3.3.1 Single dose

To account for variation in kidney function the popPK compartment model was refitted after including CrCl as a premultiplier to *Cl/F*. In this model it is the (*Cl/F*)‐to‐CrCl ratio rather than *Cl/F* that is estimated by the corresponding fixed effect. The estimated average (*Cl/F*)‐to‐CrCl ratio in a population with mean *k*_*a*_ meeting eligibility criteria was 17.4 (95% CI [13.5, 21.3]).

AIC and BIC for this model were −48.9 and −30.6, while for the model without CrCl they were, respectively, −57.0 and −38.7. As ‘smaller is better’, these criteria preferred the model without CrCl. Nevertheless, estimates of *k*_*a*_ and *K* from the two models were quite similar (Table A3) and residual diagnostics indicated the CrCl model was still a good fit, hence we proceeded to use it to estimate dose recommendations for various levels of CrCl (Section 3.3.3).

#### 3.3.2 Repeat dose

**Figure 2:**
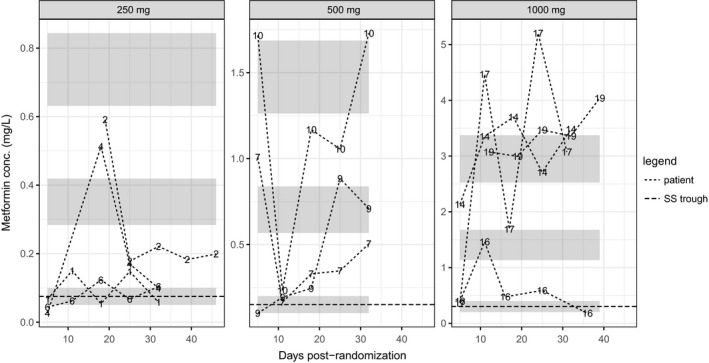
Repeat‐dose serum metformin concentrations recorded at Visits 3‐6 for each dose group 24 hours postdose (trough levels) for those patients with *more than* one observation above the upper limit of the 95% CI for the mean of the steady‐state trough level (patients 1, 2, 4, 6, 7, 9, 10, 14, 16, 17, and 19). Number labels and lines indicate observed value; from top to bottom, ribbons show the 95% CIs for the means of the theoretical steady‐state maximum, mean, and trough concentrations, respectively, for a population meeting eligibility criteria with average Cl/F and *k*_*a*_. Note that different vertical scales are used in each panel to allow the plots to be easily read

**Figure 3:**
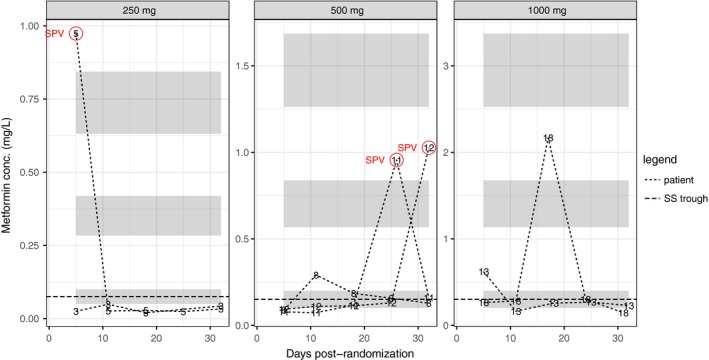
Repeat‐dose serum metformin concentrations recorded at Visits 3‐6 for each dose group 24 hours post‐dose (trough levels) for those patients with *no more than* one observation above the upper limit of the 95% CI for the mean of the steady‐state trough level (patients 3, 5, 8, 11, 12, 13, and 18). Number labels and lines indicate observed value; from top to bottom, ribbons show the 95% CIs for the means of the theoretical steady‐state maximum, mean, and trough concentrations, respectively, for a population meeting eligibility criteria with average Cl/F and *k*_*a*_. Note that different vertical scales are used in each panel to allow the plots to be easily read

**Figure A1:**
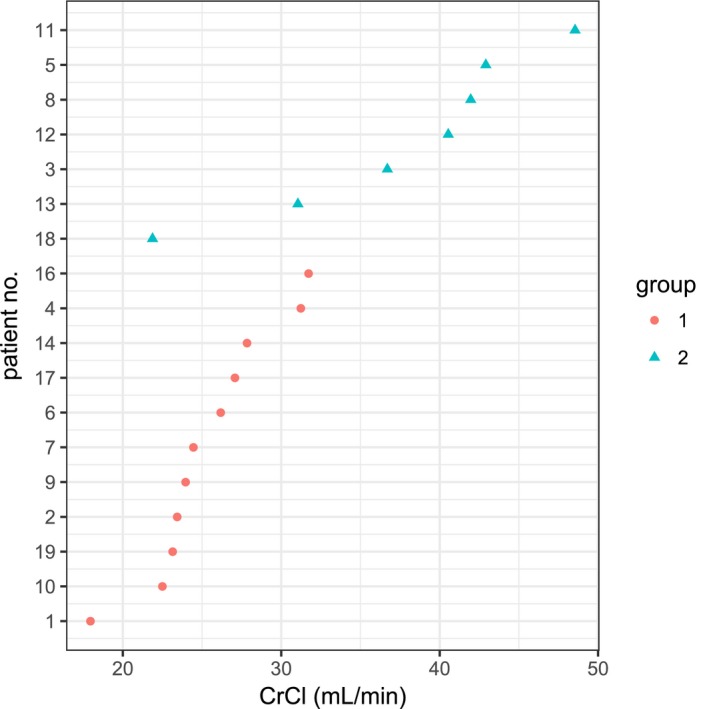
Creatinine clearance at baseline by group as defined in Section 3.3.2

To explore the relationship between CrCl and trough concentrations, we split the study cohort into two groups by comparing observed trough levels at visits 3‐6 with steady‐state minimum concentrations implied by the popPK model (Equation (1)) fitted to the single‐dose data. Patients with more than one observed trough concentration above the upper limit of the 95 percent confidence interval for the steady‐state minimum were classified as Group 1 (see Figure 2), the remainder as Group 2 (Figure 3). There were 11 patients in Group 1 and 7 in Group 2. Three of the 6 observations above the upper limit of the 95% confidence interval in Group 2 were suspected protocol violations (see Section 2.4.4), but were not excluded from any analyses. Median CrCl at baseline in Group 1 was 24 mL/min (IQR 4.18 mL/min) and in Group 2 was 41 mL/min (IQR 8.57 mL/min; see Figure A1). The difference was significant at the 0.05 level (Wilcoxon *W *=* *12, *P *=* *0.0154); a nonparametric estimate for the difference between the groups was 14 mL/min (non‐parametric 95% CI: [5.0, 19]). The maximum CrCl in Group 1 was 32 mL/min (patient 16). The minimum in Group 2 was 22 mL/min (patient 18), however, all the rest were 31 mL/min or greater.

#### 3.3.3 Dose recommendations

The popPK compartment model with CrCl can be used to estimate repeat‐dose peak concentrations for a given dose and baseline CrCl. They may be useful in determining the dose required to obtain a given steady‐state concentration for a patient with known baseline CrCl. Estimates are given for CrCl 30‐50 mL/min in Table A4. On average, for patients meeting our eligibility criteria and having mean *Cl/F* and *k*_*a*_, and CrCl = 30 mL/min, the estimated *c*_max,ss_s for 250, 500, and 1000 mg daily doses are 0.7 mg/L (95% CI: [0.58, 0.82]), 1.4 mg/L (95% CI: [1.17, 1.64]), and 2.81 mg/L (95% CI: [2.33, 3.28]).

## 4 DISCUSSION AND CONCLUSIONS

We conducted the largest consecutive dose‐escalating study of metformin in patients with metformin‐naïve T2DM and CKD‐4 to date. Eighteen patients with eGFR < 30 mL/min/1.73 m^2^ were allocated to 1 of 3 dose groups: 250 mg/day, 500 mg/day, and 1000 mg/day. Administration was oral by immediate‐release tablet. We fitted a single‐compartment popPK model to 102 concentrations (5‐6 per patient) post–first dose. Bardin et al^27^ and Doung et al^15^ (instant release formulation) also modeled metformin concentration in patients with T2DM and impaired renal function and similarly found a single‐compartment model to be appropriate.

The fixed effects parameters in our single‐compartment model were the absorption and elimination rate constants (*k*_*a*_ and *K*) and the apparent clearance (*Cl/F*). Fixed effect estimates for AUC_0‐∞_, *t*_max_, *C*_max_, and the absorption and elimination half‐lives ($t_{1/2}^{k_{a}}$ and $t_{1/2}^{K}$) were derived using the delta method (Table 2). Patient‐specific random intercepts were added for *Cl/F* and *k*_*a*_ to account for the repeated measurements on patients.

### 4.1 Single‐dose pharmacokinetics

Time to maximum concentration (*t*_max_) and maximum concentration at *t*_max_ (*C*_max_) were consistent with previous observations in patients with normal renal function (healthy and diabetic). Our estimate for mean *t*_max_ was 3.19 hours (95% CI: [2.58, 3.80]); those for mean *C*_max_ by dose were 250 mg: 0.69 mg/L (95% CI: [0.59, 0.78]); 500 mg: 1.37 mg/L (95% CI: [1.18, 1.57]); and 1000 mg: 2.74 mg/L (95% CI: [2.35, 3.13]). In their review Graham et al^2^ estimated mean *t*_max_ to be about 3 hours and *C*_max_ between 1.0 and 1.6 mg/L for a 500 mg dose.

Sambol et al^12^ give means from two groups with chronic renal impairment after an oral dose of 850 mg. Renal function in their patients was similar to ours (moderate impairment group: CrCl 31‐60 mL/min, n = 5; severe impairment group: CrCl 10‐30 mL/min, n = 6). Mean *t*_*max*_s were 3.75 hours and 4.01 hours; the latter being larger than the upper limit of our confidence interval but comparable with empirical median *t*_*max*_s observed in our 500 mg and 1000 mg groups (4.0 hours in both; see ref.^16^ Table 2). Sambol et al's^12^ mean *C*_max_s were 4.12 mg/L and 3.93 mg/L, higher than our modeled (see above) and empirical median *C*_max_s for comparable doses (500 mg: 1.13 mg/L; 1000 mg: 2.28 mg/L). It remains unclear why Sambol et al^12^ observed higher *C*_max_s. However, due to small group sizes and differences in study cohorts, sampling variation should not be ruled out. Metformin naïveté was not stipulated and, unlike our cohort, 7 of their moderate or severe patients were nondiabetic. Moreover, mean ages were 45.5 years (SD: 6.1; moderate) and 38.3 (SD: 13.6; severe) compared with a mean of 64.1 years (SD: 7.9) for our 500 mg and 1000 mg patients.

Tucker et al^32^ give means from 2 groups with T2DM, but better renal function after a single 1000 mg oral dose (“Group II”: CrCl 85‐120 mL/min n = 4; “Group III”: CrCl 51‐116 mL/min n = 8). With mean *t*_*max*_s of 2.1 hours and 2.4 hours and corresponding mean *C*_max_s of 3.25 mg/L and 3.24 mg/L their patients reached higher maximum concentrations in shorter times than our patients.

Oral clearance (*Cl/F*) under our eligibility criteria was estimated to be on average 494 mL/min (95% CI [397, 591]). The upper limit is well below mean *Cl/F* for patients with normal renal function (CrCl > 80 mL/min) which is estimated to be 1140 mL/min (SD = 330).^2^ Between‐patient variation is reflected in the range of our best linear unbiased predictors (BLUPs) which was 178‐799 mL/min. Tucker et al's^32^ T2DM groups had means 947 mL/min and 718 mL/min; near the upper end of the range of our BLUPs. Sambol et al's^12^ renally impaired groups had means 238 and 259 mL/min; at the lower end of the range. This is consistent with the fact that clearance of metformin decreases in proportion with CrCl^2^, ^12^ and both our cohort and Sambol et al's^12^ had lower CrCl than Tucker et al's^32^

In a second popPK model we fitted *Cl/F* as a constant multiple of patient‐specific baseline CrCl. We estimated the average (*Cl/F*)/CrCl ratio to be 17.4 (95% CI: [13.5, 21.3]); the range of the BLUPs was 5.7‐32.1. Graham et al's^2^ estimate for the population average was 10.7 (SD = 3.5). Duong et al^2^ analyzed data from a group composed of healthy patients (n = 185), patients with T2DM (n = 98), and patients with CKD (n = 22). They estimated the median (*Cl/F*)/CrCl ratio to be 12.3 and the range to be 5.6‐42.5. Our estimates seem consistent with both albeit slightly higher than those of Graham et al^2^ The (*Cl/F*)/CrCl ratio does not appear to be dependent on CrCl as our range of BLUPs is similar to Duong et al's^15^ range which was based mostly on healthy patients and we observed no evidence of a trend in plots of patient‐specific ratios against CrCl.

### 4.2 Repeat dose

Patients with observed trough levels within the range predicted by the popPK model fitted only to the 24‐hour concentrations had significantly higher CrCl at baseline than patients whose observed trough concentrations were consistently higher than the predicted range. Graham et al^2^ also found that steady‐state predictions from first‐order compartment models were consistent with observed trough concentrations in healthy and diabetic patients with mild renal impairment (mean CrCl 83 mL/min). Our results suggest that this fails to hold when renal impairment becomes severe, that is, at CrCl below about 32 mL/min. Below this level, metformin concentrations should be monitored.

Our estimated steady‐state average and maximum concentrations at 500 mg/day were 0.70 mg/L (95% CI: [0.57, 0.84]) and 1.47 mg/L (95% CI: [1.26, 1.69]), both higher than those found in healthy patients by Timmins et al^17^ : 0.35 mg/L and 0.65 mg/L for average and maximum, respectively. However, those authors used extended‐release tablets while we used immediate release. Duong et al^15^ reported a steady‐state average concentration among healthy subjects of 0.9 mg/L (range 0.6‐1.1 mg/L) and 1.28 mg/L (range 0.2‐7.7 mg/L) among patients with T2DM. However, these results are difficult to compare with ours because, as noted above, Duong et al's^15^ T2DM cohort was more heterogeneous; *Cr/Cl* ranged from 15 to 127 mg/L (median 67 mL/L); and daily metformin doses ranged from 250 to 3000 mg (median 1500 mg).

### 4.3 Dose recommendations

It remains unclear what the therapeutic range of metformin is in patients with impaired renal function. Frid et al^14^ proposed an upper limit of 2.8 mg/L while Duong^15^ used 5 mg/L. Graham et al^2^ suggest the average steady‐state concentration rather than the maximum is most clinically relevant for dosing. Duong et al^15^ suggested that 500 mg/day taken in immediate‐release tablets would likely keep maximum concentration under 5 mg/L for patients with CrCl = 15 mL/min.

The steady‐state concentrations implied by our popPK model appeared to be unreliable for CrCl below 30 mL/min so we give dose recommendations for values above this cutoff only. For patients meeting our eligibility criteria, having mean *Cl/F* and *k*_*a*_, our estimated average *c*_*max,ss*_ for patients with CrCl = 30 mL/min at the maximum dose of 1000 mg/day was 2.81 mg/L (95% CI: [2.33, 3.28]). Therefore, at this dose, according to Frid et al's^14^ criterion, patients with these characteristics should be closely monitored.

Standard model comparison criteria (AIC and BIC) indicated the model without CrCl was a better fit to the data than the model with it. However, due to our modest sample size and observation that the compartment model breaks down at low CrCl values we would not interpret this as evidence that metformin clearance is unrelated to CrCl.

### 4.4 Conclusion

This is the largest phase I pharmacokinetic trial yet performed in patients with CKD. The single‐dose PK parameters *t*_*max*_ and *C*_*max*_ were consistent with previous observations in patients with normal renal function (healthy and diabetic). The association between CrCl and apparent clearance (*Cl/F*) of metformin was also similar to that observed in patients with normal renal function. However, *Cl/F* itself was much lower than in healthy patients and correspondingly steady‐state minimum concentrations implied by our popPK model were higher.

Model‐based steady‐state concentrations appeared to fit the data well among a group of patients with high CrCl (median CrCl 41 mL/min), but not among a group with low CrCl (median CrCl 24 mL/min), suggesting that the first‐order compartment model with absorption in a peripheral compartment breaks down as CrCl declines. We were probably able to detect this because, relative to previous studies, a much larger proportion of our study cohort (56%) had very low CrCl (<30 mL/min). Therefore, while the results suggest that 500‐1000 mg per day could be taken by CKD‐4 patients, metformin levels should be monitored regularly.

## DISCLOSURE

None declared.

## APPENDIX 1

### 1.1

**Table A1:** eGFR and CrCl values at baseline for all patients. CrCl is reported for adjusted, ideal, and total body weight

Patient no.	Dose (mg/day)	Gender	Height (m)	Weight (kg)	BMI (kg/m^2^)	eGFR (mL/min/1.73 m^2^)	CrCl (mL/min)		
							Adjusted BW^b^	Ideal BW^a^	Total BW
1	250	F	1.45	94.7	45.04	17	17.96	11.39	27.81
2		M	1.72	117.3	39.65	15	23.43	17.46	32.4
3		M	1.74	129.3	42.71	27	36.7	28.92	48.37
4		F	1.74	128.1	42.31	21	31.24	19.47	48.88
5		M	1.68	83.3	29.51	21	42.91	30.67	61.27
6		M	1.73	77.2	25.79	22	26.18	19.9	35.6
7	500	M	1.83	126.1	37.65	21	24.46	21.02	29.61
8		M	1.8	122.2	37.72	29	41.96	36.12	50.72
9		M	1.62	86.7	33.04	16	23.96	20.06	29.82
10		M	1.68	82.2	29.12	18	22.5	17.34	30.23
11		M	1.72	124.9	42.22	29	48.55	36	67.36
12		M	1.71	111.8	38.23	29	40.54	30.33	55.86
13	1000	M	1.62	134.9	51.4	29	31.05	27.64	36.16
14		M	1.8	149.8	46.23	23	27.84	26.52	29.82
16		M	1.66	111.5	40.46	19	31.73	25.32	41.33
17		M	1.68	89.7	31.78	17	27.08	21.63	35.25
18		F	1.77	101.5	32.4	18	21.87	17.64	28.22
19		M	1.76	105.4	34.03	20	23.15	20.73	26.77

a IBW: For men = 50 + 2.3 × (height (inches)‐60) and for women = 45.5 + 2.3 × (height (inches)‐60).

b If BMI < 18.5 kg/m^2^ value calculated using TBW; if BMI 18.5‐22.9 kg/m^2^ value calculated using IBW; if BMI ≥ 23 kg/m^2^ value calculated using ABW, where ABW = IBW + 0.4 × (TBW‐IBW).

**Table A2:** Regression of dose and baseline CrCl on log metformin concentrations 24 hours postdose (trough levels)

Parameter	Unit	Estimate	SD error	DF	*P*‐value	95% CI
Intercept	—	−0.92	0.74	70	0.219	(−2.40, 0.56)
Dose 500	mg	1.42	0.45	14	0.007	(0.45, 2.39)
Dose 1000	mg	2.36	0.45	14	<0.001	(1.40, 3.32)
CrCl	L/h	−0.87	0.38	14	0.037	(−1.68, −0.06)
CrCl (standardized)^a^	SD(CrCl)	−0.30	0.13	14	0.037	(−0.58, −0.02)
*sd(ran. eff)* b	—	0.67	—			(0.41, 1.09)
*Resid. SE* c	—	0.82	—			(0.70, 0.97)

a Coefficient after standardizing log metformin and CrCl.

b Standard deviation of the patient‐specific random intercept.

c Within‐patient standard error.

**Table A3:** Compartmental pharmacokinetic parameters of metformin administered in daily doses of 250, 500, and 1000 mg to patients with CKD‐4. Estimates and predictions from the model with CrCl

Parameter	Unit	Fixed effects^a^		BLUP Summaries^b^	
		Estimate	95% CI	Median	Range
*K*	1/h	0.128	(0.113, 0.143)		
*k*_*a*_	1/h	0.564	(0.401, 0.729)	0.56	(0.03, 1.0)
$t_{1/2}^{K}$	h	5.42	(4.78, 6.06)		
$t_{1/2}^{k_{a}}$	h	1.23	(0.88, 1.58)		
*Cl/Fʹ* c	L/h	17.4	(13.5, 21.3)	16.0	(5.7, 32.1)
*t*_max_	h	3.40	(2.82, 3.98)		
		Random effects			
sd(Cl/F*ʹ*)^d^	mg/L	7.81	(5.41, 11.26)		
sd(k_a_)	1/hr	0.31	(0.20, 0.47)		
cor(Cl/F*ʹ*,k_a_)		0.79	(0.49, 0.93)		
Resid. SE^e^	mg/L	0.99	(0.08, 0.12)		

a Fixed effects estimates and CIs are for the average value over a population of patients meeting eligibility criteria and with average Cl/F*ʹ* and *k*_*a*_. Variation in population averages is typically lower than variation in observations on individual patients.

b BLUPs are best linear unbiased predictors for the study cohort. They indicate the variation in observed values for the study cohort. They are only given for the random effects Cl/F*ʹ* and *k*_*a*_.

c *Cl/Fʹ* is the (Cl/F)/CrCl ratio.

d Standard deviation of the patient‐specific random effects for Cl/F*ʹ* and *k*_*a*_ and their correlation.

e Within‐patient standard error.

**Table A4:** Estimated average repeat‐dose serum metformin concentrations, and 95% CIs, at doses 250**‐**500 mg for baseline values of CrCl 40**‐**50 mL/min

CrCl mL/min	Dose (mg)	C_min,ss_ (mg/L)		C_avg,ss_ (mg/L)		C_max,ss_ (mg/L)	
		Est.	95% CI	Est.	95% CI	Est.	95% CI
30	250	0.06	(0.04, 0.09)	0.33	(0.26, 0.41)	0.70	(0.58, 0.82)
35		0.06	(0.03, 0.08)	0.28	(0.22, 0.35)	0.60	(0.50, 0.70)
40		0.05	(0.03, 0.07)	0.25	(0.19, 0.30)	0.53	(0.44, 0.61)
45		0.04	(0.03, 0.06)	0.22	(0.17, 0.27)	0.47	(0.39, 0.55)
50		0.04	(0.02, 0.05)	0.20	(0.16, 0.24)	0.42	(0.35, 0.49)
30	500	0.13	(0.08, 0.18)	0.66	(0.52, 0.81)	1.40	(1.17, 1.64)
35		0.11	(0.07, 0.15)	0.57	(0.44, 0.69)	1.20	(1.00, 1.41)
40		0.10	(0.06, 0.13)	0.50	(0.39, 0.61)	1.05	(0.87, 1.23)
45		0.09	(0.05, 0.12)	0.44	(0.35, 0.54)	0.94	(0.78, 1.09)
50		0.08	(0.05, 0.11)	0.40	(0.31, 0.49)	0.84	(0.70, 0.98)
30	1000	0.26	(0.16, 0.35)	1.33	(1.04, 1.62)	2.81	(2.33, 3.28)
35		0.22	(0.14, 0.30)	1.14	(0.89, 1.39)	2.40	(2.00, 2.81)
40		0.19	(0.12, 0.27)	1.00	(0.78, 1.22)	2.10	(1.75, 2.46)
45		0.17	(0.11, 0.24)	0.89	(0.69, 1.08)	1.87	(1.55, 2.19)
50		0.15	(0.10, 0.21)	0.80	(0.62, 0.97)	1.68	(1.40, 1.97)

The confidence intervals given are intervals for the mean of the steady‐state minimum, average, and maximum for those with mean Cl/F and *k*_*a*_ in the population meeting our eligibility criteria.
